# Multiple granulomatous lung lesions in a patient with Epstein-Barr-virus-induced mononucleosis and new-onset systemic lupus erythematosus: a case report

**DOI:** 10.1186/1752-1947-6-191

**Published:** 2012-07-09

**Authors:** Aki Sakurai, Shinichi Shimizu, Shinichiro Morioka, Tetsuo Fujita, Ryogo Ema, Yoshihiro Miki, Kazuhiro Tomita, Toru Nakamura, Futoru Toyoda, Yoshiro Otsuki, Hiroshi Kobayashi, Hidenori Nakamura

**Affiliations:** 1 Department of Pulmonary Medicine, Seirei Hamamatsu Hospital, 2-12-12 Sumiyoshi, Hamamatsu, Shizuoka, Japan; 2 Department of Pathology, Seirei Hamamatsu Hospital, 2-12-12 Sumiyoshi, Hamamatsu, Shizuoka, Japan; 3 Department of Thoracic Surgery, Seirei Hamamatsu Hospital, 2-12-12 Sumiyoshi, Hamamatsu, Shizuoka, Japan; 4 Department of Pathology, Tachikawa Medical Center General Hospital, 3-2-11 Kanda, Nagaoka, Niigata, Japan

**Keywords:** Centrilobular micronodules, Epstein-Barr virus, Granulomatous lesions, Lung, Sjögren’s syndrome, Systemic lupus erythematosus

## Abstract

**Introduction:**

Granulomatous lesions are commonly encountered abnormalities in pulmonary pathology, and often pose a diagnostic challenge. We report an unusual case of granulomatous lung disease with uncommon characteristics, which developed following Epstein-Barr-virus-induced mononucleosis and new-onset systemic lupus erythematosus. We aim to highlight a diagnostic approach for the condition and to raise awareness of the possibility of it being related to the immunological reaction caused by Epstein-Barr virus infection.

**Case presentation:**

A 36-year-old Japanese man, who had been diagnosed with Epstein-Barr-virus-induced infectious mononucleosis, new-onset systemic lupus erythematosus, and secondary Sjögren’s syndrome three weeks previously, presented to our facility with fever and diffuse pulmonary infiltrates. A computed tomography scan of the chest revealed multiple small nodules in both lungs. Fiberoptic bronchoscopy with bronchoalveolar lavage revealed lymphocytosis with predominance of T lymphocytes. A histological examination of a lung biopsy taken during video-assisted thoracic surgery showed randomly distributed tiny granulomatous lesions with infiltration of eosinophils. The differential diagnoses included hypersensitivity pneumonitis, sarcoidosis, and pulmonary involvement of Crohn’s disease, systemic lupus erythematosus, and Sjögren’s syndrome, but the clinical and pathological findings were not consistent with any of these. Our patient’s condition did not improve; therefore, prednisolone therapy was started because of the possibility of specific immunological reactions associated with Epstein-Barr virus infection. After steroid treatment, our patient showed radiological and clinical improvement.

**Conclusions:**

To the best of our knowledge, this is the first case of a patient developing randomly distributed multiple granulomatous lung lesions with eosinophilic infiltrates after Epstein-Barr virus infection and systemic lupus erythematosus. On the basis of our data, we hypothesize that Epstein-Barr virus infection altered the immune response of our predisposed patient and contributed to the pathogenesis of the lung lesions. Our patient’s clinical response to steroid treatment was excellent.

## Introduction

Granulomatous lesions are commonly encountered abnormalities in pulmonary pathology. Most granulomas in the lung are caused by mycobacterial or fungal infections; however, if no organisms are detected, then it is necessary to consider non-infectious causes of granulomatous disease such as sarcoidosis, hypersensitivity pneumonitis, hot tub lung, Wegener’s granulomatosis, and Churg-Strauss syndrome [1].

We report an unusual case of granulomatous lung disease that differed from the above-mentioned entities, which developed following Epstein-Barr virus (EBV)-induced mononucleosis and new-onset systemic lupus erythematosus (SLE) in our patient.

EBV has been linked to SLE for 40 years, and a growing body of experimental evidence supports a possible role for EBV in the etiology of SLE [2,3], but the clear mechanism of this linkage has not been elucidated. Although there have been several case reports linking EBV and SLE [4,5], to the best of our knowledge this is the first case of a patient who developed multiple eosinophilic granulomatous lesions in the lung after the diagnosis of new-onset SLE following EBV-induced mononucleosis.

This case report highlights a diagnostic approach for granulomatous lung disease and aims to raise awareness of the possibility of the connection between granulomatous lung disease and immunological reaction caused by EBV.

## Case presentation

A 36-year-old Japanese man, with a history of presumed Crohn’s disease at age 16 years, on the basis of peri-anal fistula with compatible histopathological features and spontaneous healing without medical intervention, presented to our facility with a 10-day history of fever (39°C to 40°C), malaise and generalized lymphadenopathy. He also had a history of photosensitivity and erythema on the cheeks and nose over the past five years, which had worsened over the previous two days. Our patient had no history of smoking or asbestos exposure. On examination, he was febrile and had tachypnea. He had a bilateral malar rash, tender cervical lymphadenopathy, swelling of parotid and submandibular glands, and arthritis affecting the bilateral metacarpophalangeal joints of the hands. He also had a dry mouth and dry eyes, which were confirmed by a positive Schirmer’s test (<5mm per five minutes, bilaterally). Auscultation of the heart and lungs was normal. His abdomen was remarkable for moderate hepatomegaly and splenomegaly.

On admission, his laboratory test results showed: leukocytosis (white blood cells 13,090 cells/μL; lymphocytes 57.4%, atypical lymphocytes 10.0%); elevated liver enzymes (alanine aminotransferase 202U/L (normal range 10 to 40U/L), aspartate aminotransferase 172U/L (normal range 13 to 30U/L), alkaline phosphatase 1332IU/L (normal range 100 to 320IU/L), γ glutamyltranspeptidase 223IU/L (normal range 5 to 60IU/L), lactate dehydrogenase 561IU/L (normal range 110 to 210IU/L)); and normal renal function and urine analysis with serum angiotensin-converting enzyme 16.4U/L (normal range 7 to 25U/L). Serum serological tests were positive for anti-nuclear antibody 1:80 (normal value <1:40), double-stranded DNA (dsDNA) antibodies 22IU/mL (normal value <10IU/mL), and anti-Sjögren syndrome antigen A (SS-A)/Ro antibody >500 (normal value <10).

Virological test results for human immunodeficiency virus antibody, cytomegalovirus antigen and antibody, hepatitis B surface antigen and antibody, hepatitis C virus antibody were negative, but our patient was positive for IgG and IgM to EBV viral capsid antigen and IgG to EBV early antigen. Our patient was negative for IgG to Epstein-Barr nuclear antigen (EBNA). A chest X-ray was clear and computed tomography (CT) revealed a small amount of pleural effusion in addition to lymphadenopathy in bilateral cervical, mediastinal and peritoneal areas, and hepatosplenomegaly. A neck lymph node biopsy showed positive expression of EBV-encoded RNA by *in situ* hybridization, and the presence of serum EBV DNA was confirmed by polymerase chain reaction (PCR). Based on our patient’s clinical course, a diagnosis of EBV-associated infectious mononucleosis, SLE and secondary Sjögren’s syndrome was made, with a SLE Disease Activity Index (SLEDAI) score of 11.

His serum lactate dehydrogenase and alkaline phosphatase levels gradually improved after three weeks of hospitalization, with no treatment. Improvement was also shown on physical examination, and our patient was discharged on day 21.

**Figure 1:**
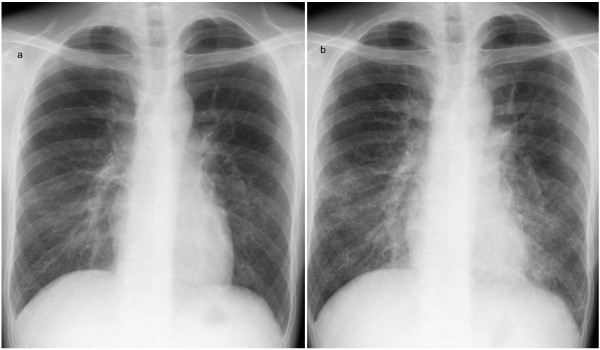
**Chest radiographs taken at our patient’s first (a) and second (b) admission.** The chest radiograph from the second admission (b) showed a bilateral interstitial pattern.

**Figure 2:**
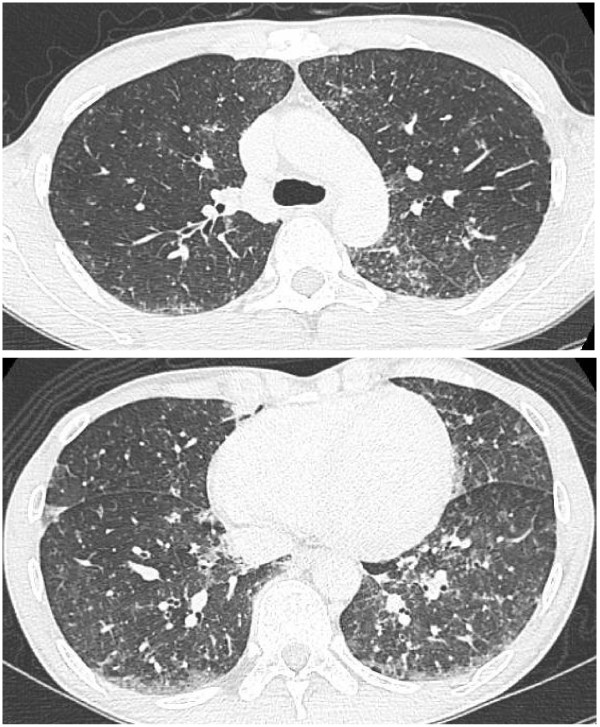
**High-resolution chest computed tomography (CT) scan taken at our patient’s second admission.** The CT scan shows diffuse, randomly distributed, ill-defined, centrilobular micronodules with patchy ground glass opacity in both lung fields.

Two weeks after initial hospitalization, he presented with high fever and mild dyspnea on exertion. Physical examination was unremarkable except for high fever (40°C) with SaO_2_ of 98% in room air. PCR results for serum EBV DNA were negative and the titers for anti-nuclear antibody (ANA) and anti-dsDNA antibodies declined to low levels, with no clinical signs of SLE exacerbation (SLEDAI score of 3). A chest X-ray revealed interstitial infiltration in both lungs (Figure 1), and a chest CT scan showed diffuse, randomly distributed, ill-defined centrilobular micronodules with patchy ground glass opacity in both lung fields (Figure 2). Pulmonary function tests revealed impaired gas diffusion with a forced vital capacity (FVC) of 129.2% of predicted values, forced expiratory volume in one second (FEV1) of 100.8% of predicted values, FEV1/FVC of 72.8%, and diffusion capacity of the lung for CO (DLCO) of 66.2% of the predicted value. Fiberoptic bronchoscopy with bronchoalveolar lavage revealed markedly increased cell counts with predominance of lymphocytes (84%) with 1% neutrophils, 1% eosinophils and 14% macrophages. Flow cytometry revealed a predominance of T lymphocytes (99%), with 35.6% CD4^+^ and 62.8% CD8^+^ cells; the ratio of CD4^+^/CD8^+^ was 0.57.

**Figure 3:**
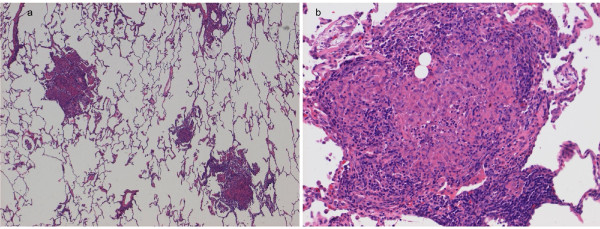
**Lung biopsy revealing non-necrotizing granulomas with infiltration of eosinophils (a, b).** Video-assisted thoracic surgical lung biopsy stained with hematoxylin and eosin ((a), ×30; (b) × 150). There were nodular lesions measuring up to 1 mm in size, containing well-formed, non-necrotizing, epithelioid cell granulomas, various types of inflammatory cell infiltration including eosinophils, and exudates of fibrin. These were randomly distributed in the central air spaces of the lung.

On the basis of our patient’s medical symptoms, the differential diagnoses included hypersensitivity pneumonitis (HP), EBV-related lymphomatoid granulomatosis, lymphocytic interstitial pneumonia secondary to Sjögren’s syndrome and SLE, and sarcoidosis. To confirm the diagnosis, a lung biopsy was obtained by video-assisted thoracic surgery, and sections from the right middle lobe revealed nodular lesions measuring up to 1 mm in size, containing well-formed, non-necrotizing, epithelioid cell granulomas, comprising lymphocytes, plasma cells, and eosinophils on hematoxylin and eosin staining, whereas peri-bronchiolar cellular infiltration and alveolitis were unremarkable (Figure 3). These lesions were randomly located within the central airspaces of the lung, and did not show bronchiolocentric distribution, which is commonly seen in patients with HP. The results of tissue stains and cultures for bacteria, mycobacteria and fungi were all negative, and EBV-encoded RNA expression in the lung was not detected by *in situ* hybridization. Immunohistochemical staining results for S-100 protein were also negative.

HP was considered as a possible etiology of our patient’s symptoms. However, our patient was not known to have been exposed to any inhaled agents, nor did he show any clinical improvement during hospitalization. In addition, the histopathological features of the lung biopsy were not typical of those of HP. The other possibilities, such as sarcoidosis, pulmonary involvement of Crohn’s disease, SLE, and Sjögren’s syndrome were also considered to be unlikely based on clinical and pathological findings.

Our patient’s condition did not improve: he had continued daily high-grade fever, and there was no improvement in his radiological findings. Thus, prednisolone therapy was started at a dose of 0.5mg/kg/day, as a result of the possibility of specific immunological reactions associated with EBV infection. Our patient showed remarkable clinical and radiological improvement, and he was discharged on a tapering dose of prednisolone.

After one year of follow-up, our patient remained asymptomatic and had no relapse after discontinuation of prednisolone. Physical examination and chest X-ray/CT results were normal. Serological studies showed an absence of ANA and anti-dsDNA antibodies, and the presence of anti-SS-A/Ro antibodies, EBNA IgG, and anti-smooth muscle antigen antibodies.

## Discussion

Recent research has revealed that EBV is a strong candidate etiological agent for SLE, based on pleiotropic interactions with host immune responses, such as molecular mimicry, adjuvant or bystander effects, and interferon (IFN)α induction [2,6,7]. Our patient had photosensitivity and butterfly rash five years before the diagnosis of SLE and his symptoms of lupus, such as pleural fluid and arthritis, resolved spontaneously after the acute phase of EBV infection. Considering the clinical course, he might have had unrecognized subclinical SLE several years before the diagnosis, and EBV infection might have influenced his immune response and played a crucial role in the pathogenesis of SLE and the onset of clinical manifestations.

In addition, our patient developed multiple tiny, well-formed granulomatous lesions in the lung three weeks after the diagnosis of primary EBV-induced infectious mononucleosis, new-onset SLE, and Sjögren’s syndrome. Infectious causes, such as tuberculosis, atypical mycobacterial infection, and fungi, were excluded based on histochemical staining, cultures, and PCR. Drug-induced pneumonitis was also excluded.

Therefore, non-infectious granulomatous lung diseases were considered in the differential diagnosis. Histological characteristics of major non-infectious granulomatous diseases are summarized in Table 1, according to the distribution and the presence of necrosis.

**Table 1:** Classification of non-infectious granulomatous lung diseases according to the distribution and the presence of necrosis

**Distribution of granuloma**	**Presence of necrosis**	**Disease**
Bronchocentric (inhalation)	Non-necrotizing	Hypersensitivity pneumonitis, lymphocytic interstitial pneumonia, talcosis
	Occasionally necrotizing	Histiocytosis X
Lymphangitic	Non-necrotizing	Sarcoidosis, chronic beryllium disease
Angiocentric	Necrotizing	Wegener’s granulomatosis, lymphomatoid granulomatosis
	Occasionally necrotizing	Churg-Strauss syndrome
Nodular	Necrotizing	Rheumatoid nodule

HP, which is a diffuse granulomatous interstitial lung disease caused by inhalation of various agents, was considered based on our patient’s radiological and histopathological findings. Although the diagnosis of HP is often difficult to make [8], in our patient HP was unlikely given the lack of a history of exposure to occupational and domestic agents, and the fact that the lung biopsy lacked the characteristic features of HP, such as alveolitis with significant lymphocytic infiltrates within the interstitium, even in areas where no granulomas are present, and poorly formed interstitial granulomas [9]. Furthermore, our patient failed to respond to removal from antigen exposure by hospitalization. Serological study results were negative for precipitating antibodies to fungal antigens, such as *Aspergillus* sp. and *Trichosporon cutaneum*.

Another possibility that we considered was sarcoidosis. Histologically, sarcoidosis is characterized by well-formed interstitial, non-caseating granulomas, which show lymphangitic distribution [10]. In our patient, the distribution was different from that of sarcoidosis, and his serum angiotensin-converting enzyme level was normal.

Crohn’s disease, which shares pathological and immunological features with sarcoidosis [11] and can develop granulomatous lung disease as extra-intestinal involvement [12], was also considered to be unlikely, because the evidence of gastrointestinal involvement of Crohn’s disease was not confirmed by upper endoscopy and colonoscopy.

Furthermore, we raised the possibility that the pulmonary manifestation was associated with SLE and Sjögren’s syndrome. Pulmonary involvement is frequently seen in SLE [13], and there is a case report of acute granulomatous lupus pneumonitis [14]*.* Sjögren’s syndrome, which shares similar clinical and radiographic features with sarcoidosis [15], is also known to demonstrate various types of pulmonary manifestations, including granulomatous infiltrations [16]. Even though SLE and Sjögren’s syndrome seemed to be in a dormant state when our patient developed pulmonary manifestations, and granulomatous lesions are not typical findings of these conditions, we cannot exclude the possibility that these lesions were attributable to SLE and Sjögren’s syndrome.

Lastly, EBV was considered as a possible etiological agent. Lymphomatoid granulomatosis, which is a rare EBV-associated lymphoproliferative disorder, was excluded histopathologically, and there have been no reports to suggest involvement of EBV in granulomatous reactions in the lung, which can be seen in the spleen, bone marrow and liver [17,18]. However, considering the fact that EBV is known to cause pleiotropic interactions with host inflammatory responses, and in the present case, played a crucial role in the development of SLE clinically, we hypothesize that EBV infection might have altered the immune response and contributed to the pathogenesis of the lung lesions through aberrant T cell responses, dysregulated IFN and other cytokines responses, and dysfunctional CD8 responses [2,3,6,7]. Rapid improvement in the symptoms by administration of prednisolone also supports an immunological process.

## Conclusions

We describe the case of a patient who developed randomly distributed multiple granulomatous lung lesions with eosinophilic infiltrates, following EBV infection and new-onset SLE. Although we cannot fully explain the linkage between these conditions, we think that the immunological reaction induced by EBV contributed to the pathogenesis of the lung lesions, as well as the onset of SLE. His clinical and radiological response to prednisolone was excellent.

## Consent

Written informed consent was obtained from the patient for publication of this case report and any accompanying images. A copy of the written consent is available for review by the Editor-in-Chief of this journal.

## Competing interests

The authors declare that they have no competing interests.

## Authors’ contributions

SS, YO and HK made substantial contributions to the histological examination and revised the manuscript. HN made substantial contributions to conception and design of the study, and analysis and interpretation of data. KT and YM were involved in revising the manuscript critically for important intellectual content. SM, TF and RE were involved in the care of our patient in the pulmonary department. TN and FT performed a thoracoscopic lung biopsy and revised the manuscript. All authors read and approved the final manuscript.
